# Collectanea Medica: Consisting of Anecdotes, Facts, Extracts, Illustrations, &c.

**Published:** 1820-01

**Authors:** 


### COLLECTANEA MEDICA:

CONSISTING OF

### ANECDOTES, FACTS, EXTRACTS, ILLUSTRATIONS, &c.

***Relating to the History or the Art of Medicine, and the Auxiliary Sciences.***

Floriferis ut apes in saltibus omnia libant,

Omnia nos itidem depascimur aurea dicta.

### *Further Observations on the Departments and Periodical Actions of Glands.*

[BORDEU, *Rechérches sur les Glandes,* § cxix.]

THE periodical actions of the glands, in a general point of view, appears to be proved by what has already been detailed ;* but many points of this subject yet remain to be elucidated. We should know,—1°, what length of time each gland is in action ; 2°, at what hour, nearly, it begins to act in the natural day ; 3°, what are the glands which are *congeneras,* or which may, and really do, act together ; 4°, what, on the contrary, are those which cannot act together, either because they mutually suspend the actions of each other, or because they should not act together, in conformity to the harmony of the system ; 5°, what are those which may act vicariously for some others which do not act ; 6°, if there are glands intended to supply in a manner the want of the actions of others, &c. &c.

It cannot be doubted that all these questions, and many others which might be made, would much elucidate our knowledge of the animal economy, if it were possible to determine them with sufficient precision.

We acknowledge that we cannot explain them : this only we will propose on the subject. Let us suppose that, after a restricted diet for two days, or about that time, a person of the best constituted, and the most regular, state of body, has slept during eight or ten hours ; the tranquil and perfect sleep has suspended all the excretions, all the actions, of the glands ;—let us make an abstraction, too, for the changes effected by the passions. The person in question is one of those sober and regular peasants who are only occupied by the immediate object of their attention ; or let it be a child.

This person rises in the morning ; he stretches himself, and *rouses up*, if we may so express it, all his members : after some minutes he begins to find an appetite for food, and then, as we have already observed, his parotids enter into action ; they continue their functions until the stomach is sufficiently full ; they then repose, or only produce as much secretion as is necessary to moisten the mouth and throat.

The stomach then, in turn, begins to enter into increased action :

* The whole of Bordeu's arguments on this subject have not been given in the previous extracts : only one or two of the most interesting points of them were chosen.—EDIT.

it is occupied in digesting the food; and soon afterwards, the fluids which it sends into the duodenum irritate this intestine; the pancreas, the liver, and the spleen, enter into play; these three viscera act with the stomach and the duodenum; they have their nerves and their vessels from the same trunks, and they apparently continue in action until the stomach and duodenum are emptied.

It is now for the small intestines to act: they will excite the large ones and the lacteal vessels, which, filling themselves, will put the mesentery in action, until all the chyle is absorbed, and until it has passed into the reservoir of Pecquet and the thoracic duct; which have also, without doubt, their particular action. If any portion of the chyle be absorbed by the originative branches of the vena porta, may not the liver be again irritated? May there not be a peculiar action of the liver, different from that which effects the secretion of the bile?

The flow of the chyle into the heart and the lungs, should excite the vessels and the powers affecting the circulation; after a certain time, the kidneys and the skin will evacuate the excrements of the digestion, &c.

What the bladder, the rectum, and the other reservoirs, receive in deposit, will escape when these reservoirs shall be excited by certain causes, &c. In what class shall we place the action of the breasts, that of the uterus, and that of the genitals?

Such appears to be nearly the arrangement and the relation of these actions, as well at least as they can be perceived in the state of perfect health. All these digestions, which may be reduced to secretions and excretions, seem to be effected in the space of about six or eight hours, the greatest part of which time is employed by the digestion in the stomach and duodenum, and in the elaboration of the chyle in the blood-vessels.

Monasteries, villages, cities even, furnish us with examples of those well-ordered people, the whole of whose functions are arranged in the most regular manner. They eat every day at the same hours; they rise, they go to rest, at marked hours; their appetite comes at fixed times; they awake, and go to sleep, at precise hours; and, what is more remarkable, is, that, if they pass the moment marked for some one of their functions, they find their system deranged until they again fall into their ordinary train.

Every person has perceived that, when the hour of repast approaches, the appetite increases; and that, if they have let it pass without eating, the appetite disappears of itself, not to return until some hours afterwards. We shall not here seek the cause of this phenomenon,* nor to examine if what has been said respecting it be well-

---

* It may be right to remark, that Bordeu never seeks for the proximate cause of any of the phenomena of the human body; he only endeavours to trace their history and relations: his imagination, and the fire by which he is carried on in his reasonings, not unfrequently leads him to mark relations that probably do not exist; but this is the only error into which he may have fallen. He did not, like LA CAZE, BARTHEZ, and some other physicians of the school of Montfounded. It is sufficient that it is itself one proof of what we have advanced respecting the marked terms for the action of certain parts.

How many persons do we not find who sweat every morning at the same hour? There are some who constantly wake at marked hours; others have certain excretions every day at periods marked out for them. I have a friend who has no other chronometer for the night than his chest: he has an expectoration come on every morning towards four o'clock, after having coughed for a few minutes. This never fails him, except when he deranges his ordinary mode of life. We see persons who vomit mucous matter every morning at nearly the same hour, &c.

We might also speak of many periodic hæmorrhages from the nose, by the anus, and from other parts. Hildanus saw a patient who voided every morning, about nine or ten o'clock, a considerable quantity of blood by the navel. We often meet with young persons who have hæmorrhages from the nose every morning for a certain time. We know what takes place with respect to hæmorrhoids, in the menstruation of women, &c.

The greater number of physiologists treat of the circulation only in a general point of view: they do not remark that it may be very different in the large and in the small vessels. Has not each organ its particular circulation, which may be augmented or diminished, without any participation of the general system?

There is some apparent evidence that the circulations are more or less *prompt* according to the different orders of vessels, and according to the action and use of different parts. These truths may be regarded as corollaries to what we have already advanced.

There is, then, a general circulation, and many particular circulations. These are, if we may venture so to express it, like *little circles* which terminate, or fall in, with a larger one. We have been accustomed to make use of this denomination of a *circle*, to express that a part, although it receives its blood by means of the *general circulation*, or that which is effected in the great vessels, has moreover a *particular circulation*, according as it is in action or is not in action. The other parts which participate this action, are of the *department of its circle*, &c.

Thus, the smallest part may be regarded as making in a manner a *distinct body*. It acts, it is true, by means of the general circulation; but it is also as distinct from it as the system of blood-vessels is from the system of chylous vessels, and as the circulations of the lungs and of the liver are from that which is effected in the ordinary great vessels.

---

pellier of the same period, in opposing the doctrine of Böerhaave, adopt any of the modifications of the hypothesis of Stahl: indeed, he expressly says, on several occasions, that the proposition of a *vital principle*, as an abstract cause of the sensible phenomena of the human body, is at least a conjecture, if not a supposition, and is therefore unfit for a basis for our reasonings.—Edit.

We have sufficiently explained what we consider to constitute health; it depends on certain connexions, order, and relations, of the organs and of their actions: many diseases, on the same basis, are only an *overthrow* of this harmony,—a disturbance of the functions, &c. These general notions are true, and generally acknowledged.

Is it possible, in any certain disease, to find out which is the faulty organ, that which deranges the actions of the rest, and how it effects this? This is not easy, because we have hitherto rested on too general ideas. May not the paroxysms of certain maladies, and their periodical returns, depend on the order of the functions of the organs? Let it be supposed that a certain organ acts every day at a certain hour, may we not suspect that it especially contributes to produce the phenomena observed at that same hour; and if there are organs whose actions encounter each other every second or every third day, may we not establish the same suspicions, and elucidate what has been so much talked of,—the crises and critical days; and distinguish what is real from what is imaginary on these matters.

It must be avowed, that what has hitherto been said on the exacerbations in continued fevers, on the paroxysms in intermittents, on the periodical returns of certain gouty and rheumatic pains, does not appear to be sufficiently well established to permit us to depend on them as certain principles: why should we, then, blame a physician who should seek routes which might lead to some discoveries respecting them.

Let us not forget to remark that, as some great practitioners have already observed, each age has its particular diseases: those of the head are very common in early life; those of the chest appear afterwards; and then those of the belly and of the extremities. It cannot be denied that there is some truth in this remark: may not these vicissitudes depend on those which arise in the action of certain organs?—and may we not say, that, as each age has its particular diseases, so each age has its favourite organs?

If each age has its organs, each person, almost, has his; and, by reducing them to certain classes, we might perhaps find what has been so much sought for respecting the *temperaments:* one person rests all his life subject to the dependencies of a certain organ, if we may be permitted so to express it; another depends on the *influences* of some other part; in this, the brain acts more, proportionally, than the stomach; in another it is the reverse; here it is the liver; there the genital organs; in another, the skin, the muscles, the membranes, &c.

All these combinations, which really exist, being reduced to distinct classes, we might determine, it seems, the temperaments; and, without stopping at generalities, which are too vague, we might advance in important knowledge.

---

From various observations in the most recent works of respectable authors, as well as from private converse with some generally well-informed physiologists, we find that the notion respecting the manner of the heart's action in the circulation of the blood that was established soon after the discovery of HARVEY, continues to be for the most part adopted; or, rather, there is not a suspicion entertained by many persons as to its truth. The point expressly alluded to here, is the dilatation of the ventricles. This is, according to that doctrine, a passive act, dependant on simple relaxation of the parietes of those cavities, and the impulse of the blood derived from the forces generally circulating it, and the contraction of the auricles. This question was much agitated soon after the discovery of the circulation, and nothing can be more extraordinary than that it subsided in the manner we have alluded to: for nothing seems better established in physiology than that the dilatation of the ventricles of the heart depends on *active expansion*, or *automatic motion*, of their parietes. Those who wish to trace this doctrine on its renewal in more modern times, are referred to the works of PLENCK, GRIMAUD, DUMAS, BICHAT, Dr. WILSON of Newcastle, ZUGENBUHLER, and Dr. CARSON.* From the treatise of Dr. Carson, we shall extract a passage describing, generally, the circulation of the blood, according to the doctrine in which the point above designated plays so important a part. The influence of this doctrine in physiological and pathological reasonings, is of the most serious kind; and a knowledge of the truth must throw a light over many interesting subjects, which, without it, must be obscure in the extreme. Indeed, the circulation of the blood, simply, cannot be satisfactorily understood on the principles generally admitted. Let those to whom this may appear conjectural (as we know it does to some persons) go into a butcher's slaughter-house, and have the heart removed from a bullock immediately on its being slain: he will find that he cannot confine it by his hands, as Bichât remarked;—the expansive power of the walls of the ventricles exceeds that of the contractile force of a man's hand, and the heart will contract and expand in spite of his efforts. For a full account of this subject, we refer to the work of Dr. Carson, in which this doctrine is demonstrated with that methodic precision only to be acquired by proceeding on the basis furnished by a knowledge of mathematical principles. It will doubtless appear strange to future generations, that this work, and the doctrine it illustrates, has, four years after its publication, excited so little attention, that the former has not yet arrived at a second edition, and that the latter is not generally known. As so long a period has elapsed since this work appeared, we must confine ourselves to a few extracts from it: but, lest by acting thus it may be thought that we give our sanction to the whole of the opinions contained in it, we must state, that we do not admit the notions of the author respecting the *elasticity* of the lungs, and its influence on the circulation of the blood. We shall, perhaps, recur to this subject at a future time. Dr. CARSON says,—

* *An Inquiry into the Causes of the Motion of the Blood: with an Appendix, in which the Process of Respiration, and its Connexion with the Circulation of the Blood, are attempted to be elucidated.* By JAMES CARSON, M.D. Physician to the Work-house, the Fever Hospital, and to the Asylum for the Pauper Lunatics, at Liverpool; and in Charge of the Military Hospital at that place. 8vo. pp. 250. Longman and Co. and Underwood, London.

"By every contraction of the ventricles a quantity of blood is projected forcibly into the great arteries. As the coats of the arteries are dilatable, these vessels give way to the impetuosity of the propelled blood; and, to a certain distance from the heart, become of a more enlarged calibre. In consequence of the stimuli derived from the blood newly admitted into the arterial cavity, and (if so bold a metaphor may be used) from the pain of distension, the irritability of the muscular fibres is excited; the contractile effort is roused; and, co-operating with the elastic, restores the artery by a rapid movement to its former dimensions. As the blood is an incompressible fluid, a quantity equal to that projected from the heart must now be displaced from that portion of the arterial system which had been dilated by the immediate impulse of the ventricles. The valves, which are stationed at the roots of the great arteries, and which are securely closed during the contraction of the first portion of the arterial system, by the retrograde movement of the blood nearest the heart, and more effectually by the suction occasioned by the synchronous dilatation of the ventricles, completely prevent the return of any fluid from the arteries into the heart; while the more ample calibre possessed by the aggregate of the vessels beyond the contracting portion, and the distensible condition of their relaxed fibres at the moment, favour its advance into a more distant portion. This portion, now distended and stimulated by the same causes by which the first had been excited, recoils vividly upon its contents; and, by the rapid resumption of its former capacity, expels from its cavity a quantity of blood equal to the addition which it had received during its dilatation. The blood, displaced by this movement from what may be termed the second portion of the arterial system, is directed into a more advanced part of it, by the different state of the coats of the vessels, and the difference between the aggregate of their calibre, before and behind the contracting portion; because, in consequence of the rapidity of the vibratory undulation, the coats of the vessels between the contracting portion and the heart are still rigid, while those beyond it are relaxed and easily dilatable; and because the outlets on this side are greater, as the aggregate of the bores of the vessels increases with the distances from the heart. The same series of actions is repeated to the ends of the arterial system. A quantity of blood, therefore, equal to that which had been projected from the heart into the arteries, is propelled from the ends of the arteries into the veins in equal times. The power of the heart may thus be said to be transmitted undiminished to the end of the arterial system.

"No data were discoverable from which the momentum of the blood, discharged from the ends of the arteries, or the quantity of motion it would generate in the venous blood, could be estimated. But, from the mass of fluid that was to be put in motion,—from the velocity with which it was known to flow,—from the form and position of the vessels containing it,—from the resistances opposed by friction, tenacity, and various other causes,—and from the phenomena attending the venous circulation,—it was concluded, with the fullest confidence, that the blood could not be circulated through the veins by the impulse it received from the arteries alone.

"In searching for the causes by which the chambers of the heart were dilated after contraction, it was ascertained, that this condition of the organ was in part to be ascribed to the form and position of its fibres, in consequence of which simple relaxation was accompanied by a certain degree of dilatation; but particularly to the supporting of a part of the atmospherical pressure that would have rested upon the convex surfaces of the heart or its envelope, by the resilient or collapsing effort of the lungs. It was urged, that the abstraction of a part of the ordinary pressure of the atmosphere, from the convex or external surface of the heart, or from the convex surface of the pericardium, was perpetual, and was therefore always ready to co-operate with the dilating faculty of the heart itself, as that was alternately renewed; and that the conjunction of these powers was fitted, during the intervals of contraction, to dilate the chambers to the utmost extent, or at least to the extent of the capacity of the pericardium.

"In consequence of the dilatation of the ventricles by the causes which have just been stated, the valves at the roots of the arterial trunks, yielding to the greater pressure from without, become securely closed, and the resumption of blood by the heart from the arteries is completely prevented; but the passage of the blood from the auricular into the ventricular cavities is not obstructed: the blood, therefore, by which the former chambers were dilated, pursues the less resisted course, and occupies the space left by the dilating ventricles. Any deficiency in the full dilatation of the ventricles, which in the healthy condition of these parts can scarcely occur, will readily be supplied by the projectile force of the contracting auricles. By the dilatation of the auricles, the valves in the auricular passages, sustaining less resistance on their internal surface, become securely closed; but, at the other openings, those by which they communicate with the venous trunks, no obstacles are interposed. The blood, therefore, in the large venous trunks, is relieved from a part of the ordinary pressure in the direction of the heart: it necessarily takes the course in which it meets with the least resistance, and continues to move in that course until the resistance is equalised by the full dilatation of the auricles.

The heart, therefore, acts at once in a two-fold capacity. By the contraction of the ventricles, it propels the blood through the arteries; and, by the dilatation of the auricles, it pumps it from the veins. It is at the same time a forcing and a suction pump.

"The structure of the veins is not fitted for raising blood through them to the heart, from parts at a distance from that organ, by suction alone; for these vessels, being very thin and pliant, would immediately collapse, and become impervious under such an influence. Other agents are required to preserve the permeability of the venous vessels, to give them, as it were, the property of rigid tubes, and to bring the blood which they contain generally within the sphere of the action of the heart. For this purpose, two causes are supposed to co-operate. The first, to which allusion has already been made in this recapitulation, is derived from the projectile power of the ventricles, transmitted by the vibrations of the arteries to the ends of the veins. This is the *vis a tergo,* so famous in the schools of medicine. We were unable to estimate the share of the venous circulation that is to be attributed to this cause; which, however, so far as it extends, is evidently well fitted to co-operate with an abstraction of resistance in front, and to preserve uninterrupted the communication between the blood in the remote parts and the heart.

"The other power, which is supposed to assist in preserving the distension of the venous vessels in opposition to the suction influence of the heart, was gravity. By the anastomoses which so generally prevail among the veins, particularly among the smaller ramifications, the system of the vena cava may be considered as a single canal. By its retiform fabric, the communication between the blood in the different branches, by the aggregate of which any portion of this canal is formed, is preserved as ready and free as if the blood had flowed in that portion in a single unpartitioned channel. The position of the vessel is fixed. At the moment in which the equilibrium between the contents of the vessel may be supposed to have been adjusted, a quantity of blood is abstracted from one end of it by the stroke of the heart, while a quantity is added to the other by the synchronous contraction of the ultimate portion of the arteries. The balance between the opposing columns of fluid is disturbed: to restore the equilibrium, deranged as the actions of the heart are renewed, a motion is generated in the blood by gravity from the ends of the veins to their roots.

"In short, the motion of the blood, while it flows in the veins, is produced by the force of the heart and arteries urging it behind; by the abstraction of a share of the atmospheric pressure from it in front, in consequence of the resiliency of the lungs interposing its influence in the intervals between the contractions of the heart, and by gravity, which is rendered available in this case by the projection of the arteries and the diastole of the auricles."

